# Comparative mitogenomic analysis of *Aposthonia borneensis* and *Aposthonia japonica* (Embioptera: Oligotomidae) reveals divergent evolution of webspinners

**DOI:** 10.1038/s41598-017-09003-9

**Published:** 2017-08-15

**Authors:** Zhi-Teng Chen, Liang Lü, Ming-Xing Lu, Yu-Zhou Du

**Affiliations:** 1 School of Horticulture and Plant Protection and Institute of Applied Entomology, Yangzhou University, Yangzhou, 225009 China; 2 Joint International Research Laboratory of Agriculture and Agri-Product Safety, The Ministry of Education, Yangzhou University, Yangzhou, 25009 China; 3 Key Laboratory of Zoological Systematics and Evolution, Institute of Zoology, Chinese Academy of Sciences, Beijing, 100101 China

## Abstract

In this study, we report the complete mitochondrial genome (mitogenome, mtDNA) of *Aposthonia borneensis* and compare it with another sequenced webspinner, *Aposthonia japonica*. The *A. borneensis* mitogenome is smaller than *A. japonica*, but the size of each gene and the A + T content of protein-coding genes (PCGs) are almost identical in the two mitogenomes. Among the PCGs, *atp6* shows the highest evolutionary rate and *cox1* the lowest. The mtDNA map in *A. borneensis* is similar to *Drosophila yakuba*, but distinctly different from *A. japonica*, which has extensive rearrangement. Phylogenetic analyses dated the divergence time of the two webspinners at ca. 103 Ma. We speculate that the most recent common ancestor (MRCA) of *A. borneensis* and *A. japonica* was divided into several geographic groups during the Pangea breakup. Geographic isolation between the Japanese islands and the continental southeastern Asia resulted in the divergent evolution of *A. borneensis* and *A. japonica*, thus generating mtDNA structural variations between the two species. Based on the phylogenetic analyses and specific distributional features, the genus *Aposthonia* was supported as non-monophyly, and we speculate that both highly rearranged and relatively conserved mitogenomes exist in other webspinners.

## Introduction

The insect order Embioptera, commonly known as webspinners, is a small, lesser-known order comprising a distinctive, monophyletic group with about 2,000 species inhabiting tropical and subtropical regions of the world^1, 2^. The distinctive synapomorphy of the webspinners is the presence of silk glands in the enlarged basal tarsomere of forelegs in nymphs and adults. Webspinners live subsocially in the silken galleries constructed with their silk glands and feed primarilyon plant debris. The unique morphological and biological features of webspinners make them a mysterious insect group that warrants further study. Phylogenetic relationships between the Embioptera and other insect orders remain unclear^3^. Recent studies almost unanimously support a sister group relationship between Phasmida and Embioptera^4–10^.

Mitochondrial genomes (mitogenomes, mtDNA) have been a hotspot for insect research with wide utility in phylogenetics, population genetics and evolutionary biology^11^. Insect mitogenomes are typically circular, double stranded DNA molecules, usually 14–20 kb in length, and contain 13 protein-coding genes (PCGs), 22 transfer RNA (tRNA) genes, two ribosomal RNA (rRNA) genes and a control region (CR)^12, 13^.

Kômoto *et al*. (2012) described the mitogenome of the webspinner *Aposthonia japonica* from Japan, which was the first published mitogenome for Embioptera^14^. However, the *A. japonica* mitogenome was incomplete and lacked the control region and several tRNA genes^14^. Tang *et al*. (2014) subsequently reported a partial mitochondrial sequence of *Aposthonia borneensis*, a webspinner species endemic to continental southeastern Asia^2, 15^. Unfortunately, the incomplete, fragmented mitogenome sequence data from the two *Aposthonia* spp. are insufficient for structural and evolutionary analyses. In this study, we report the complete mitogenome of *A. borneensis* and compare the organization and sequence composition with *A. japonica*. The evolutionary rate of PCGs and the mitogenome rearrangements in webspinners are discussed, and we offer speculations based on the results of phylogenetic analyses. This study is a good supplement for the limited molecular data available for Embiopterans.

## Results and Discussion

### Mitogenome organization and composition

**Figure 1:**
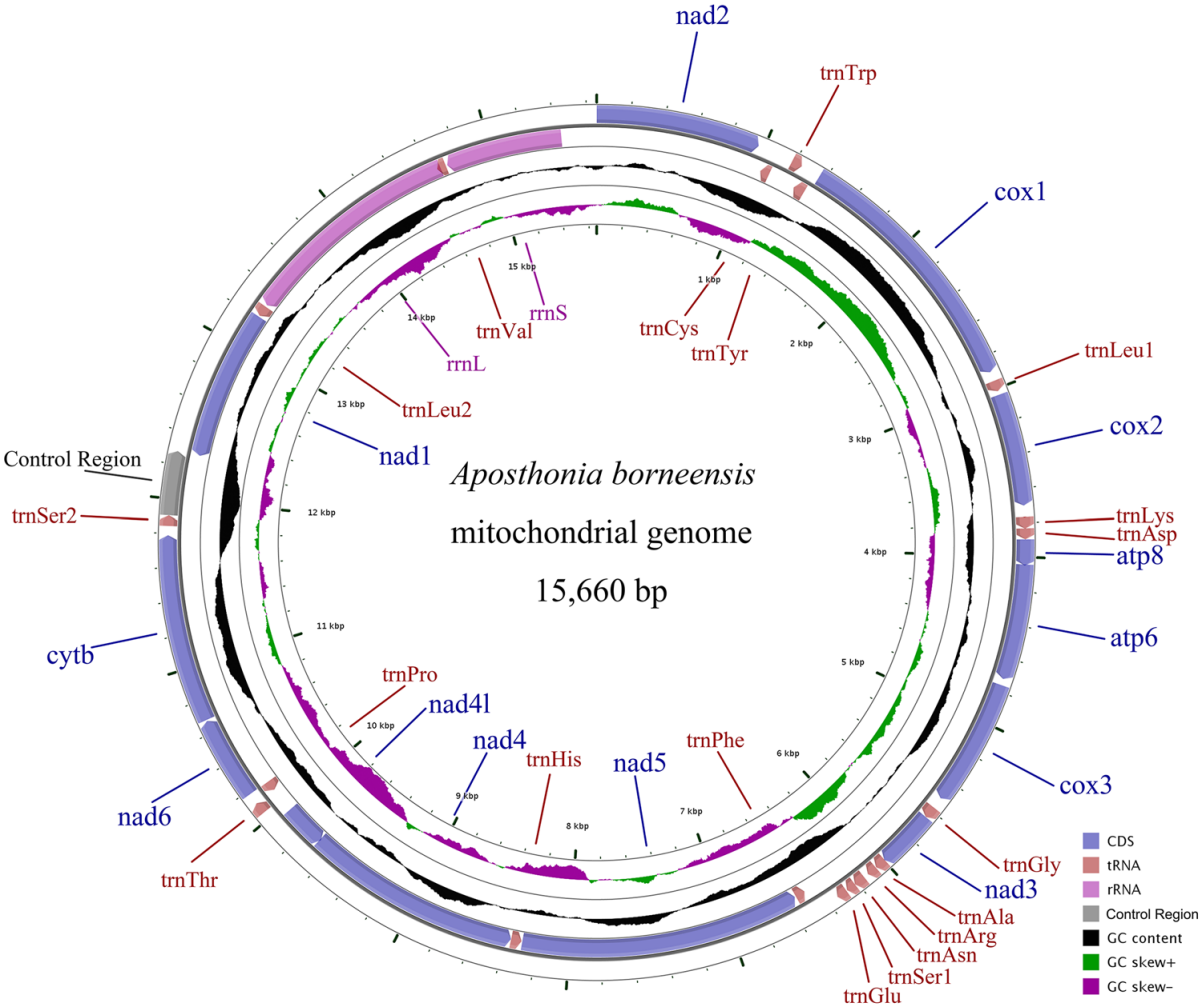
Mitochondrial map of A. borneensis. Genes outside the map are transcribed in a clockwise direction, whereas those inside the map are transcribed counterclockwise. The second circle shows the GC content and the third shows the GC skew. GC content and GC skew are plotted as the deviation from the average value of the entire sequence.

The complete mitogenome of *A. borneensis* was annotated and deposited in the NCBI database (GenBank accession no. KX965988). The *A. borneensis* mitogenome is a 15,660 bp circular DNA molecule, which is smaller than *A. japonica* (18,305 bp) due to differences in the size of intergenic nucleotides (IGNs) and the CR (Fig. 1). The A + T content of the *A. borneensis* mitogenome (75.9%) is similar to that of *A. japonica* (74.8%) (Table 1). The *A. borneensis* mitogenome contains 34 genes (13 PCGs, 19 tRNAs and two rRNAs) and a putative control region (CR); 21 genes (nine PCGs and 12 tRNAs) are located on the J-strand and 13 genes (four PCGs, seven tRNAs, and two rRNAs) map to the N-strand (Table 2). When compared to the partial mitochondrial sequences reported by Tang *et al*. (2014)^15^, the *A. borneensis* mitogenome in this study obtained more genes (*atp8*, *Cytb*, *nad6*, *rrnL*) and the CR, which ensures more reliable structural and phylogenetic analyses. The *A. japonica* mitogenome contains 13 PCGs, 20 tRNAs and three rRNAs. Three tRNAs, *trnIle*, *trnGln* and *trnMet*, are absent in both the *A. borneensis* and *A. japonica* mitogenomes. Although *trnCys* and *trnSer2* are present in the *A. borneensis* mtDNA, these genes were not reported in the *A. japonica* mitogenome, possibly because of incomplete sequence data^14^.

**Table 1:** Nucleotide composition in conserved regions of webspinner mitogenomes.

Species	Whole genome		PCGs		*rrnL*		*rrnS*		Control Region	
	Length (bp)	AT (%)	Length (bp)	AT (%)	Length (bp)	AT (%)	Length (bp)	AT (%)	Length (bp)	AT (%)
*A. japonica*	18,305	74.8	11,041	72.4	1,305	76.6	676/ 756	76.6/ 75.5	N/A	N/A
*A. borneensis*	15,660	75.9	10,966	73.3	1,376	80.0	723	75.7	377	89.9

**Table 2:** Gene structure of the mitogenome of *Aposthonia borneensis*.

Gene	Position (bp)	Length (bp)	Direction	Intergenic nucleotides (bp)	Anti- or start/stop codons	A + T Content (%)
*nad2*	1–966	966	Forward	217	ATA/TAA	76.3
*trnCys(C)*	1034–1090	57	Reverse	67	GCA	82.5
*trnTrp(W)*	1181–1243	63	Forward	90	TCA	80.9
*trnTyr(Y)*	1262–1330	69	Reverse	18	GTA	82.6
*cox1*	1362–2897	1536	Forward	-8	ATG/TAA	67.7
*trnLeu1(UUR)*	2953–3016	64	Forward	55	TAA	73.5
*cox2*	3039–3701	663	Forward	22	ATC/TAA	72.3
*trnLys(K)*	3768–3834	67	Forward	71	CTT	68.6
*trnAsp(D)*	3833–3896	64	Forward	−2	GTC	90.6
*atp8*	3897–4049	153	Forward	0	ATT/TAA	81.7
*atp6*	4043–4723	681	Forward	−7	ATG/TAA	75.3
*cox3*	4761–5510	750	Forward	0	ATA/TAA	71.3
*trnGly(G)*	5578–5649	72	Forward	67	TCC	91.7
*nad3*	5650–5997	348	Forward	0	ATT/TAA	73.9
*trnAla(A)*	5996–6061	66	Forward	-2	TGC	72.8
*trnArg(R)*	6064–6123	60	Forward	2	TCG	85.0
*trnAsn(N)*	6152–6219	68	Forward	28	GTT	80.8
*trnSer1(AGN)*	6220–6276	57	Forward	0	GCT	79.0
*trnGlu(E)*	6282–6344	63	Forward	5	TTC	85.8
*trnPhe(F)*	6494–6554	61	Reverse	149	GAA	80.3
*nad5*	6562–8289	1728	Reverse	7	ATA/TAG	75.8
*trnHis(H)*	8295–8359	65	Reverse	5	GTG	76.9
*nad4*	8361–9698	1338	Reverse	1	ATG/TAA	74.9
*nad4l*	9692–9958	267	Reverse	-7	ATG/TAA	74.9
*trnThr(T)*	10046–10114	69	Forward	87	TGT	88.4
*trnPro(P)*	10115–10176	62	Reverse	0	TGG	72.6
*nad6*	10180–10674	495	Forward	3	ATA/TAA	78.8
*Cytb*	10682–11788	1107	Forward	7	ATG/TAA	70.2
*trnSer2(UCN)*	11843–11903	61	Forward	54	TGA	86.9
CR	11904–12280	377		0		89.9
*nad1*	12281–13214	934	Reverse	0	ATG/T-	72.9
*trnLeu2(CUN)*	13222–13288	67	Reverse	7	TAG	76.1
*rrnL*	13301–14676	1376	Reverse	12		80.0
*trnVal(V)*	14655–14711	57	Reverse	−22	TAC	77.2
*rrnS*	14721–15443	723	Reverse	9		75.7

The *A. borneensis* mtDNA contains 48 overlapping nucleotides that are 2–22 bp in length and located in six pairs of neighboring genes. The longest overlap (22 bp) is located between *rrnL* and *trnVal*. A total of 983 IGNs ranging from 1 to 217 bp are dispersed between 22 pairs of neighboring genes; the longest IGN is located between *rrnS* and *nad2* (Table 2). The *A. japonica* mitogenome contains 3013 bp of IGNs, and the largest IGN (979 bp) is located between *trnThr* and *rrnSer1*. The extremely large number of IGNs reveals the relaxed structure of the two webspinner mitogenomes.

**Figure 2:**
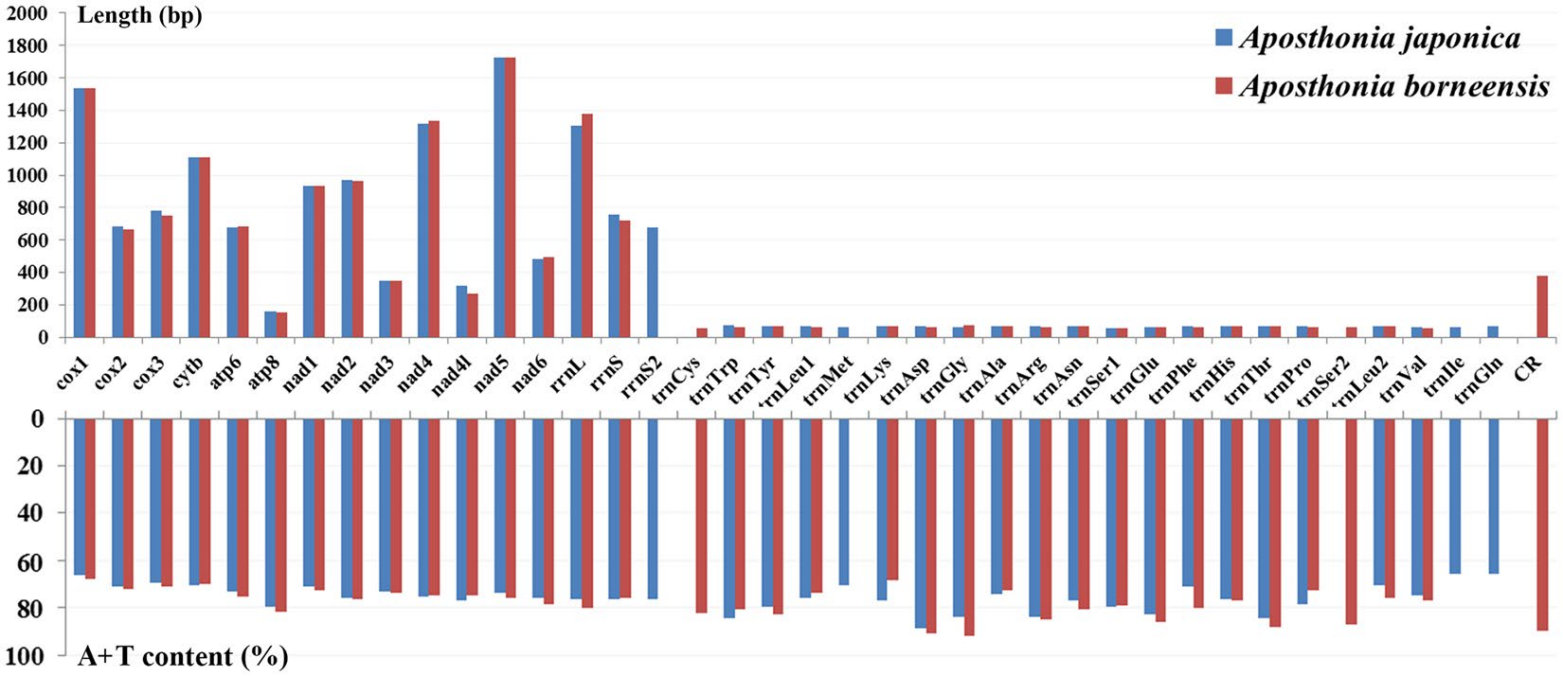
Gene size and A + T content of genes in the two webspinner mitogenomes. *A. japonica* and *A. borneensis* are represented by blue and red columns, respectively. Gene names are shown on the X axis; gene sizes and A + T content are shown on Y axis.

The nucleotide base composition of the *A. borneensis* mitogenome (A = 43.6%, T = 32.3%, C = 17.1%, G = 7.0%) is similar to that of *A. japonica* (A = 42.5%, T = 32.3%, C = 18.7%, G = 6.5%); both genomes are significantly biased for the A and T nucleotides. The overall length and A + T content of the PCGs are similar in the two mitogenomes (Table 1). Comparative analyses indicate that the length of individual genes and the A + T content of PCGs are almost identical in the two species; a slight variability was observed in the A + T content of tRNA genes (Fig. 2). The AT-skew and GC-skews of the *A. borneensis* mitogenome were calculated and showed a bias for A and C nucleotides (Table 3). Unlike the strand bias of most other insects (positive AT- and negative GC-skew for the J-strand), the second codon position for PCGs (J-strand) shows a negative skew in *A. borneensis* ^16^.

**Table 3:** Nucleotide composition of the *Aposthonia borneensis* mitogenome.

Regions	Nucleotides Proportions (%)						AT Skew	GC Skew
	A	T	G	C	A + T	G + C		
Whole genome	43.6	32.3	7.0	17.1	75.9	24.1	0.15	−0.42
Protein coding genes	42.1	31.2	7.9	18.8	73.3	26.7	0.15	−0.41
1^st^ codon position	46.8	27.2	9.4	16.6	74.0	26.0	0.26	−0.28
2^nd^ codon position	33.8	34.9	10.3	21.0	68.7	31.3	−0.02	−0.34
3^rd^ codon position	45.6	31.6	4.1	18.7	77.2	22.8	0.18	−0.64
Protein coding genes-J	36.7	35.6	8.8	18.9	72.3	27.7	0.02	−0.36
1^st^ codon position	40.9	28.5	13.6	17.0	69.4	30.6	0.18	−0.11
2^nd^ codon position	21.3	45.2	10.6	22.9	66.5	33.5	−0.36	−0.37
3^rd^ codon position	47.9	33.0	2.4	16.7	80.9	19.1	0.184	−0.75
Protein coding genes-N	50.4	24.4	6.5	18.7	74.8	25.2	0.35	−0.48
1^st^ codon position	56.1	25.1	2.8	16.0	81.2	18.8	0.38	−0.70
2^nd^ codon position	53.3	18.7	9.8	18.2	72.0	28.0	0.48	−0.30
3^rd^ codon position	41.9	29.3	6.9	21.9	71.2	28.8	0.18	−0.52
tRNA genes	44.1	36.6	7.2	12.1	80.7	19.3	0.09	−0.25
tRNA genes-J	45.1	37.0	8.0	9.9	82.1	17.9	0.10	−0.11
tRNA genes-N	42.2	36.1	5.7	16.0	78.3	21.7	0.08	−0.47
rRNA genes	46.6	31.9	5.6	15.9	78.5	21.5	0.19	−0.48
Control region	49.6	40.3	1.1	9.0	89.9	10.1	0.10	−0.78

### Protein-coding genes

The length and A + T content of PCGs in the *A. borneensis* and *A. japonica* mitogenomes were nearly identical (Fig. 2). In both two mitogenomes, *nad5* is the largest PCG whereas *atp8* is the smallest; *atp8* has the highest A + T content while *cox1* has the lowest. Most PCGs in the two webspinner mitogenomes initiate with the standard start codon ATN (ATA, ATT, ATC, and ATG); an exception is *nad4* in the *A. japonica* mitogenome, which initiates with GTG. Most of the PCGs have complete termination codons (e.g. TAA or TAG), whereas *nad1* contains the incomplete termination codon T for the two mitogenomes (Table 2). Ojala *et al*. (1981)^17^ speculated that the incomplete termination codon T could be completed by post-transcriptional polyadenylation.

**Figure 3:**
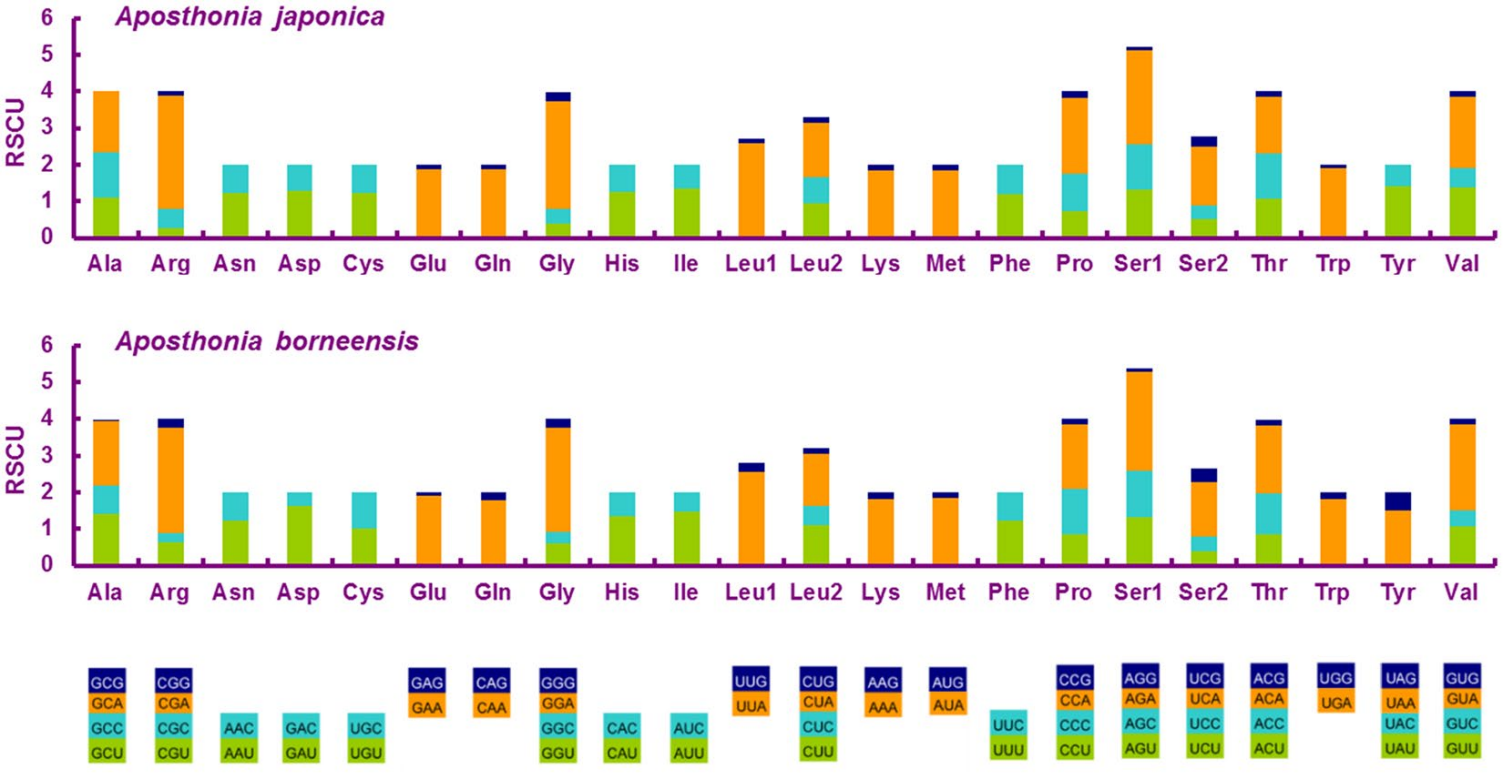
Relative synonymous codon usage (RSCU) in the two webspinner mitogenomes. Codon families are indicated below the X axis.

In the *A. borneensis* mitogenome, the AT-skew of the concatenated PCGs is positive whereas the GC-skew is negative, indicating that the concatenated PCGs contain a higher percentage of AT vs. GC nucleotides. AT-skews were positive at the first and third positions but negative at the second position of the combined PCGs (Table 3). The relative synonymous codon usage (RSCU) values of the two mitogenomes were compared and showed a biased use of A and T nucleotides in both webspinners (Fig. 3). The frequent use of anticodons NNA and NNU reflects the preference for A and T in the third codon position. Overall, the five most frequently used codons in *A. borneensis* and *A. japonica* are ATA (Met), ATT (Ile), AAA (Lys), AAT (Asn) and TTA (Leu), which further supports the strong bias for A and T nucleotides.

**Figure 4:**
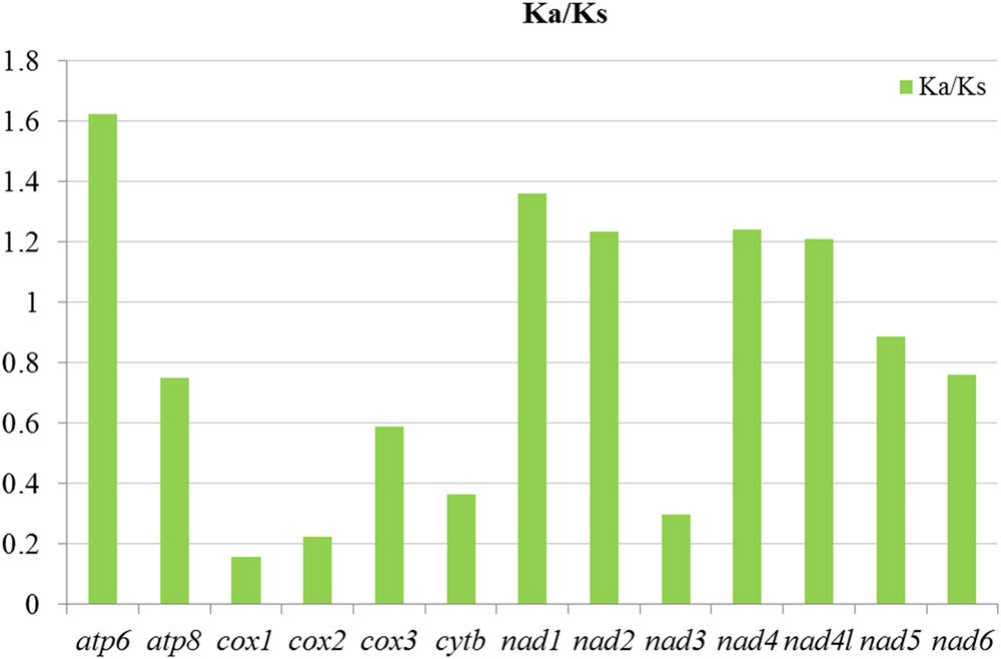
Evolutionary rates of PCGs in the two webspinner mitogenomes. The green columns indicate the average Ka/Ks for each gene.

The mean Ka/Ks ratios for individual PCG of the two webspinner mitogenomes are calculated to evaluate the evolutionary rate of each PCG (Fig. 4). The results indicate that *atp6* has the highest evolutionary rate, followed by *nad1*, while *cox1* has the lowest rate. The Ka/Ks ratios for *atp6*, *nad1*, *nad2*, *nad4* and *nad4l* were all above 1, indicating that these five genes are evolving under positive selection. The Ka/Ks ratios for the remaining eight PCGs were all below 1, suggesting the existence of high evolutionary pressure.

### Transfer RNA and ribosomal RNA genes

Unlike the typical set of 22 tRNA genes in metazoan mitogenomes, there are 19 and 20 tRNA genes predicted in the *A. borneensis* and *A. japonica* mtDNAs, respectively. The size of tRNA genes was conserved in the two species, but the A + T content varied in the tRNA genes of the two mitogenomes. In the *A. borneensis* mitogenome, 14 tRNA genes are predicted to fold into typical cloverleaf secondary structures composed of four arms; however, the remaining five tRNAs lacked dihydrouridine (DHU) arms (Supplementary Fig. S1). A total of 20 mismatched nucleotides (G-U pairs) were identified in the *A. borneensis* tRNAs. In general, the anticodons of the 19 tRNA genes in *A. borneensis* are essentially identical to those reported in other insects.

**Figure 5:**
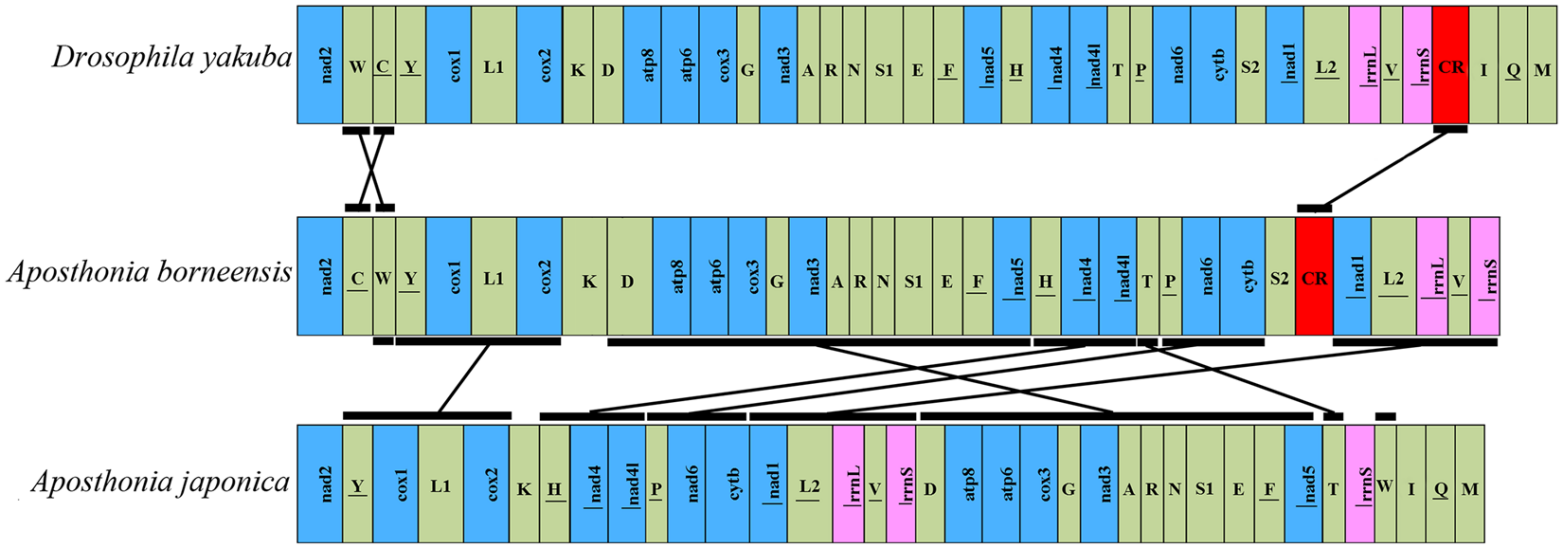
Mitogenome arrangements in *Drosophila yakuba*, *A. borneensis*, and *A. japonica*. Genes are transcribed from left to right except for the underscored genes, which are transcribed in the opposite direction. Rearrangements are indicated by the diagonal black lines.

In the two webspinners, the size of the large ribosomal RNA (*rrnL*) genes ranged from 1305 (*A. japonica*) to 1376 bp (*A. borneensis*), and the A + T content of *rrnL* ranged from 76.6% (*A. japonica*) to 80.0% (*A. borneensis*) (Table 1). In both species, the two *rrnL* genes mapped between *trnLeu2* and *trnVal*, which is common in other insect mitogenomes (Fig. 5).

The small ribosomal RNA (*rrnS*) gene of *A. borneensis* is 723 bp with an A + T content of 75.7%. In *A. japonica* two *rrnS* genes were identified, one gene is 756 bp and located adjacent to the *rrnL* gene, while the other copy is 676 bp and maps between *trnThr* and *trnTrp*. Kômoto *et al*. (2012)^14^ speculated that the 676 bp copy is a pseudogene due to its truncated 3′ end.

### The control region

**Figure 6:**
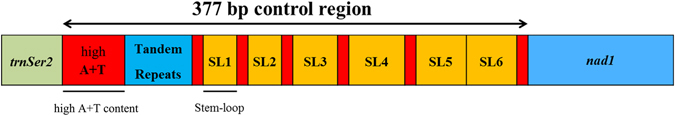
Predicted structural elements in the control region of *A. borneensis*. The regions with high A + T content are indicated by red shaded rectangles. Stem-loop regions are demarcated by orange rectangles.

The non-coding CR was not identified in the *A. japonica* mitogenome, presumably due to incomplete sequencing^14^. In *A. borneensis*, the putative CR is 377 bp, has an A + T content of 89.9%, and is located between *trnSer2* and *nad1* (Table 2). The unusual location of the CR in *A. borneensis* is presumably due to mtDNA rearrangement. Structural elements in the CR of *A. borneensis* can be predicted beginning at the 5′ end: a 66-bp region with high A + T content (90.9%); a 44-bp region containing two complete and one incomplete tandem repeats; and six stem-loop structures (SL) (Fig. 6). The small size of tandem repeats may explain the relatively small CR in *A. borneensis* when compared to other insects^18, 19^. The tandem repeats and SL structures in the CR have potential regulatory effects on mitogenome replication and transcription^20^.

### Gene rearrangements

The mtDNA map is generally conserved in insects and similar to the model insect, *Drosophila yakuba* ^21^; however, exceptions exist and some insect groups exhibit rearranged mitogenomes. Analysis of the mtDNA sequences of the two webspinners revealed gene rearrangements in both species. In *A. borneensis*, a translocation is present between *trnCys* and *trnTrp*, and the putative CR mapped between *trnSer2* and *nad1*, which is also presumably due to translocation (Fig. 5). In general, the organization of the *A. borneensis* mitogenome is highly similar to *D. yakuba*. However, the mitogenome of *A. japonica* differs from *D. yakuba* and shows multiple rearrangements including an extra copy of *rrnS* and the translocation of *trnTrp*, *trnThr*, the *trnAsp*-*nad5* gene cluster, and perhaps *trnCys* and *trnSer1* (Fig. 5). Several hypotheses for these rearrangements were discussed by Kômoto *et al*. (2012)^14^, but the underlying mechanisms remain unclear.

The arrangement of mitochondrial genes is generally conserved among closely related species; consequently, the magnitude of structural variations in the two *Aposthonia* spp. is noteworthy. This phenomenon is very rare for animals but has been recorded for booklice in the genus *Liposcelis* ^22^ and has been found recently in a thrips genus^23^.

Several hypotheses have been proposed to explain the heightened mitogenome rearrangement rates in insect species. One hypothesis considers parasitism as a predisposing factor for mtDNA reorganization, but this has been gradually rejected^24, 25^. Another hypothesis is concerned with the duplication of the control region, which has been confirmed in the Thysanoptera and Psocoptera^26–28^, but does not apply to webspinners. Another theory is related to the evolution of haplodiploidy, which has been reported in Hymenoptera, Thysanoptera, Aleyrodidae, Sciaridae but does not apply to webspinners^29^. Although the underlying mechanisms for mtDNA rearrangement in insects are not clear, these rearrangements are very informative with respect to insect genesis and evolution^30^. More comprehensive analyses of insect mitogenomes in multiple insect taxa are needed to further understand the mechanistic basis of mtDNA rearrangements.

### Speculations on phylogeny and evolution

**Figure 7:**
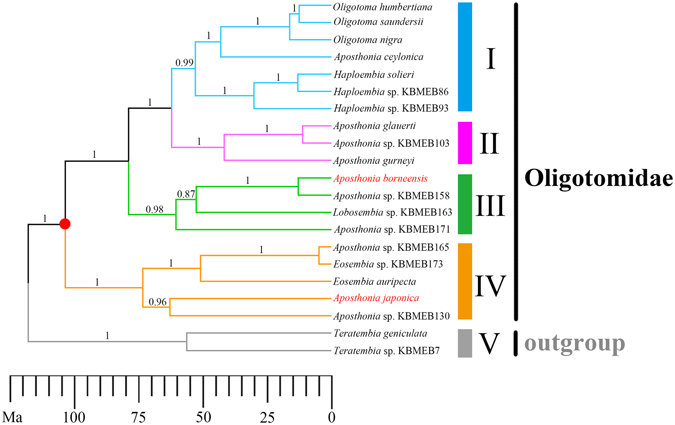
Dated Bayesian tree of Oligotomidae. Numbers on branches are Bayesian posterior probabilities. The tree was rooted with two outgroups, *T. geniculata* and *Teratembia* sp. KBMEB7.

**Figure 8:**
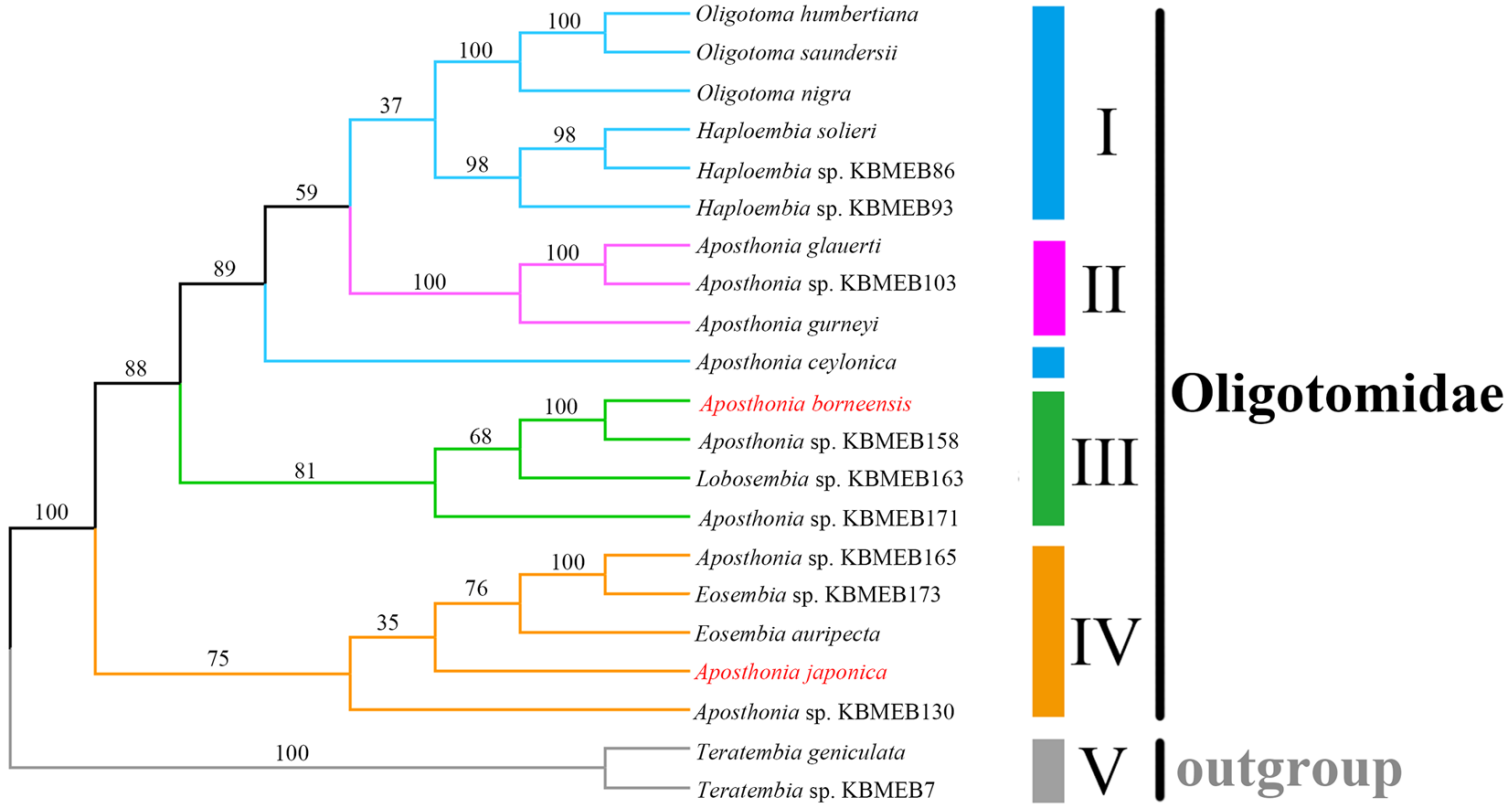
Maximum likelihood (ML) tree of Oligotomidae. Numbers on branches indicate bootstrap values. The tree was rooted with two outgroups, *T. geniculata* and *Teratembia* sp. KBMEB7.

In this study, phylogenetic analyses were conducted using the concatenated DNA sequence data of *rrnL*, 28* s*, *cox1* and *H3* genes from 19 Oligotomidae spp., and two *Teratembia* spp. from Teratembiidae were included as outgroups (Supplementary Table S1). The Bayesian inference (BI) and Maximum likelihood (ML) analysis generated similar topological structures (Fig. 7 and 8, Supplementary Fig. S2). The non-monophyly of the genus *Aposthonia* is supported in this study, which is congruent with the results of Miller *et al*. (2012)^9^. Meanwhile, the divergence time for *A. borneensis* and *A. japonica* was estimated at ca. 103 Ma (mid-Cretaceous), when the Pangea was in process of breakup. According to Blakey (2006)^44^, the Japanese islands had already been separated from the continental southeastern Asia at ca. 105 Ma. Based on these results, we speculate that the most recent common ancestor (MRCA) of *A. borneensis* and *A. japonica* was divided into several geographic groups during the Pangea breakup. Geographic isolation between the Japanese islands and the continental southeastern Asia accelerated the speciation process of the MRCA and the divergent evolution of the two webspinners. The continuous geographic isolation resulted in the genetic diversity observed between the two webspinners. For example, *A. borneensis* retained the conserved ancestral mitogenome, while a high substitution rate and frequent genetic rearrangements occurred in *A. japonica*.

In the phylogenetic trees, Oligotomidae can be divided into four clades of species according to their general distributional patterns (Figs 7 and 8). Earlier diverged clades of species (clades III and IV) tend to have a limited distribution in Asia, while later-diverged clades of species (clades I and II) show a farther distribution away from Asia (Supplementary Table S1). This result should be a good evidence for our speculation that divergent evolution of webspinners accompanied the Pangea breakup, and the species of Oligotomidae might originate from Asia. Furthermore, the five PCGs with relatively high evolutionary rates (*atp6*, *nad1*, *nad2*, *nad4* and *nad4l*) may also have potential influence on this divergent evolutionary process. Finally, based on phylogenetic analyses, we predict that other webspinners exist with highly rearranged or relatively conserved mitogenomes like *A. japonica* and *A. borneensis*, respectively. Additional mitogenome sequencing of Embioptera is needed to investigate the processes driving the structural variation and evolutionary history of webspinners.

## Materials and Methods

### Sampling and DNA extraction

Specimens of *A. borneensis* were collected from South China Agricultural University in Guangdong Province, China. Our research activities were not banned by any organization or individual and did not involve endangered or protected species. Specimens used in this study were preserved in 100% ethanol and stored at −20 °C. Total genomic DNA was extracted from adults using the Column mtDNAout kit (Tianda Beijing, China) and stored at −20 °C until used for PCR.

### PCR amplification and sequencing

Five pairs of LA-PCR primers were used to amplify segments of the *A. borneensis* mitogenome (Supplementary Table S2). LA-PCR was performed with LA Taq DNA polymerase (Takara, Japan) in an ABI thermal cycler as follows: initial denaturation at 94 °C for 3 min, followed by 35 cycles at 94 °C for 30 s; annealing at 55 °C for 30 s; and elongation at 68 °C for 9 min, and final elongation at 68 °C for 15 min. LA-PCR products were purified with an Axygen DNA Gel Extraction Kit (Axygen Biotechnology, Hangzhou, China) after separation by electrophoresis in 1.0% agarose gels.

Ten additional gene-specific PCR primers were designed using Primer Premier 5.0 and used to amplify the remaining gaps (Supplementary Table S2). These reactions were performed using templates obtained from LA-PCR as follows: primary denaturation at 94 °C for 3 min, 35 cycles at 94 °C for 30 s, annealing at 40–55 °C for 30 s, elongation at 72 °C for 90 s, and final elongation at 72 °C for 10 min. PCR products were separated by 1.0% agarose gel electrophoresis, and all PCR fragments were sequenced after separation and purification.

### Genome assembly and annotation

The software CodonCode Aligner (http://www.codoncode.com/aligner/) was used for sequence assembly and annotation. PCGs and rRNA genes were identified by MITOS (http://mitos.bioinf.uni-leipzig.de/index.py) and by comparison with the previously sequenced webspinner mitogenome^14, 31^. The graphical map of the complete mitogenome was drawn with the online software CGView Server (http://stothard.afns.ualberta.ca/cgview_server/index.html)^32^. tRNAs were identified by MITOS combined with tRNAscan-SE Search Server v. 1.21 (http://lowelab.ucsc.edu/tRNAscanSE/)^33^. The secondary structure of tRNA and rRNA genes was also obtained from MITOS. The A + T content and base composition were analyzed by MEGA v. 6^34^. Composition skew analysis was performed using the AT-skew = [A–T]/[A + T] and GC-skew = [G–C]/[G + C] formulas^35^. The software package DnaSP v. 5.10^36^ was used to calculate the synonymous substitution rate (Ks) and the nonsynonymous substitution rate (Ka). Stem loop structures of the putative control region were predicted by DNAMAN, and the tandem repeats were analyzed with the Tandem Repeats Finder program (http://tandem.bu.edu/trf/trf.advanced.submit.html)^37^. Sequence data were deposited into GenBank as accession number KX965988.

### Phylogenetic Analysis and Divergence

To investigate the phylogenetic relationship between *A. borneensis* and *A. japonica*, nucleotide sequences of *rrnL*, *28s*, *cox1* and *H3* genes for 19 Oligotomidae spp. were downloaded from GenBank (Supplementary Table S1). Two other webspinners from the family Teratembiidae, *Teratembia geniculata* and *Teratembia* sp. KBMEB7 were used as outgroups. The four genes were respectively aligned with Clustal X as implemented in MEGA v. 6^34^. The best partitioning schemes and substitution models were determined with PartitionFinder v. 2.1.1^38^ using the Bayesian Information Criterion (BIC) and a greedy search algorithm. Four partitions (*rrnL*, *28* *s*, *cox1* and *H3* partitioned by genes) and the GTR + I + G model of nucleotide substitution were predetermined for analyses. Bayesian inferences (BI) were implemented in the program BEAST v. 1.8.4 on an.xml file produced in the program BEAUti v. 1.8.4 from the concatenated DNA sequence data, and the MRCA of Oligotomidae was calibrated with a lower limit of 94.3 Ma^39^. The BEAST analyses (uncorrelated lognormal relaxed clock, Yule prior) were performed for 100 million generations with sampling every 1000 generations and a burn-in of 25% trees. Stationarity of all runs was examined by Tracer v. 1.5 (Effective sample sizes over 200)^40^. Treeannotator v. 1.8.4 was used to obtain the maximum clade credibility (MCC) timetree before viewing in FigTree v. 1.4.2^41^. Maximum likelihood (ML) analysis was performed using RAxML v. 8.2.9 via the online CIPRES Science gateway portal^42, 43^. The ML analyses performed 1000 bootstrap replicates with the GTRGAMMA substitution model used for all partitions.

## Electronic supplementary material

Supplementary Information
