# Supplementary material for: Comparative mitogenomic analysis of Aposthonia borneensis and Aposthonia japonica (Embioptera: Oligotomidae) reveals divergent evolution of webspinners
Source: Sci Rep. 2017 Aug 15;7:8279. doi: 10.1038/s41598-017-09003-9 (PMC5557954; doi:10.1038/s41598-017-09003-9)
Supplement: Supplementary file 1 — Supplementary Information [file 41598_2017_9003_MOESM1_ESM.pdf]

Comparative mitogenomic analysis of *Aposthonia borneensis* and *Aposthonia japonica* (Embioptera: Oligotomidae) reveals divergent evolution of webspinners

Zhi-Teng Chen, Liang Lü, Ming-Xing Lu, Yu-Zhou Du

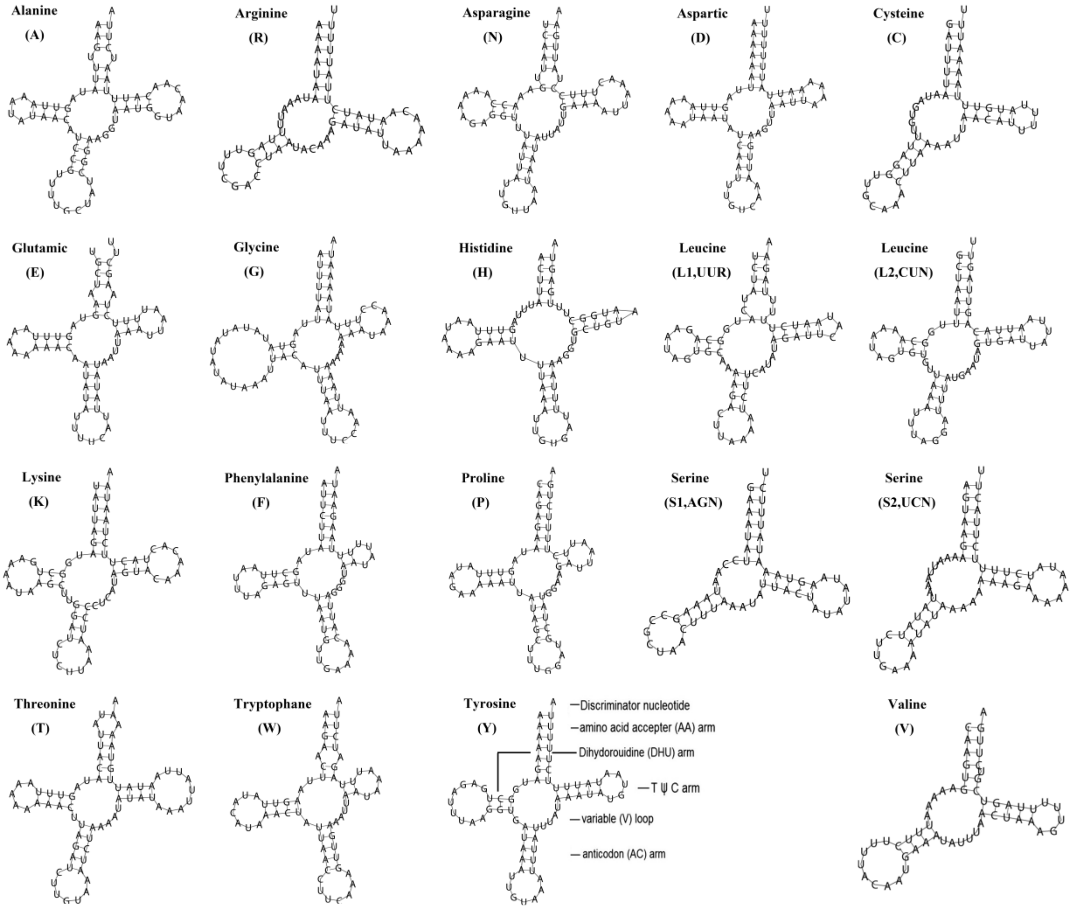

Supplementary Figure S1. Inferred secondary structures of 19 tRNAs from the *A. borneensis* mitogenome. The tRNAs are labelled with the abbreviations of their corresponding amino acids. Structural aspects in tRNA arms and loops are illustrated for *trnY*.

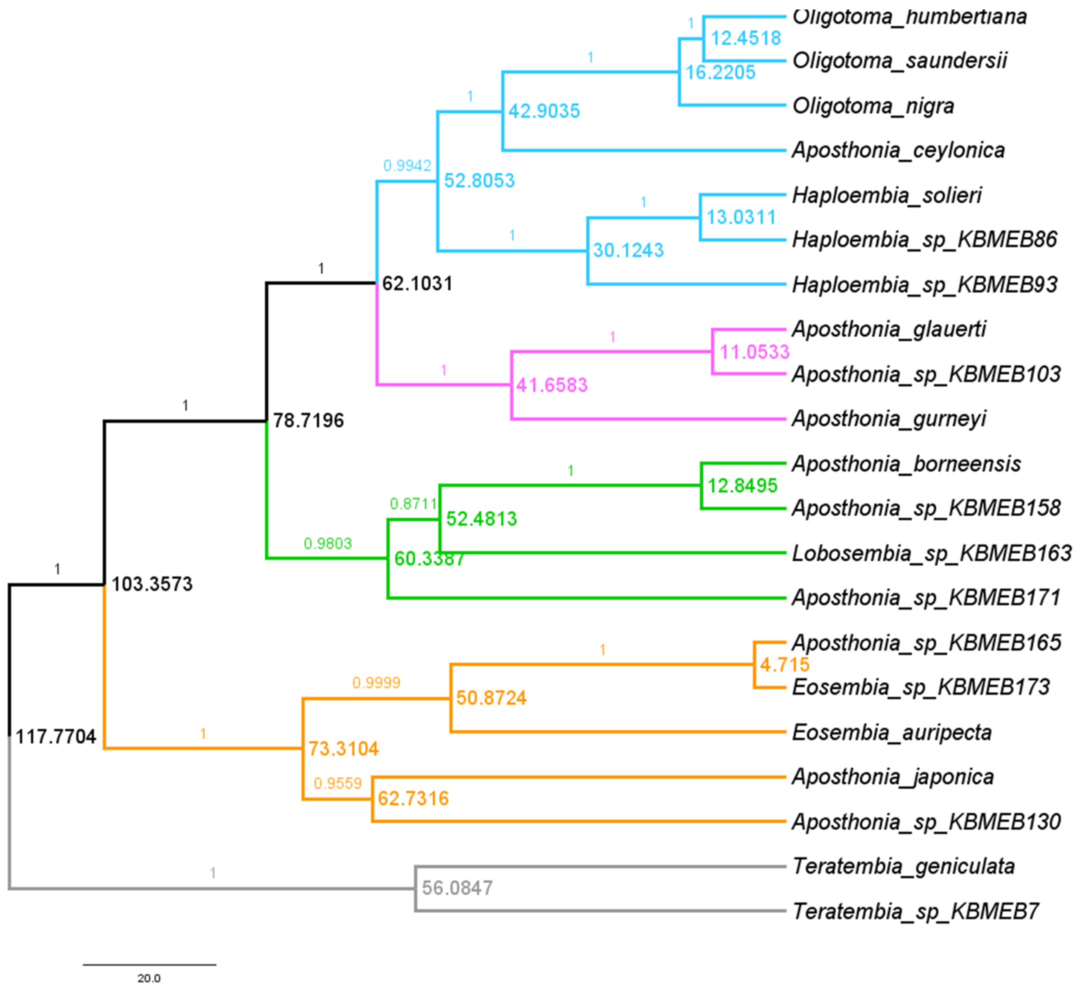

Supplementary Figure S2. Dated Bayesian tree of Oligotomidae with node ages shown. Numbers on branches are Bayesian posterior probabilities, node ages are shown at the nodes. The tree was rooted with two outgroups, *T. geniculata* and *Teratembia* sp. KBMEB7.

Supplementary Table S1. Species and sequences used in this study.

Family	Species	Locality	GenBank Accession Number			
			rrnL	***28s***	***cox1***	***H3***
	Aposthonia borneensis	Thailand, Hom Pok	JQ907184	JQ907017	JQ907083	JQ907128
	Aposthonia japonica	Japan, Kagoshima	AB639034	N/A	AB639034	N/A
	Aposthonia ceylonica	India, Karnataka	JQ907157	JQ906988	JQ907051	JQ907105
	Aposthonia glauerti	Australia, New South Wales	JQ907162	JQ906993	JQ907056	JQ907109
	Aposthonia gurneyi	Australia, Northern Territory	JQ907159	JQ906990	JQ907053	JQ907107
	Aposthonia sp. KBMEB103	Australia, New South Wales	JQ907174	JQ907006	JQ907069	JQ907118
	Aposthonia sp. KBMEB130	Malaysia, Sarawak	JQ907176	JQ907008	JQ907071	JQ907119
	Aposthonia sp. KBMEB158	Thailand, Inthanon	JQ907186	JQ907019	JQ907084	JQ907130
	Aposthonia sp. KBMEB165	Thailand, Dol Suthap	JQ907190	JQ907023	JQ907087	N/A
Oligotomidae	*Aposthonia* sp. KBMEB171	Thailand, Lumpini	JQ907193	JQ907026	N/A	JQ907135
	Eosembia auripecta	Thailand, Songkhla	JQ907191	JQ907024	JQ907088	JQ907133
	Eosembia sp. KBMEB173	Thailand, Huay Nam Dang	JQ907194	JQ907027	N/A	JQ907136
	Haploembia solieri	USA, California	JQ907149	JQ906976	JQ907039	JQ907098
	Haploembia sp. KBMEB86	Italy, Pisa	N/A	JQ907002	JQ907065	JQ907115
	Haploembia sp. KBMEB93	USA, California	JQ907172	JQ907005	JQ907068	JQ907117
	Lobosembia sp. KBMEB163	Thailand, Dol Pha Hom Pak	JQ907188	JQ907021	JQ907086	JQ907131
	Oligotoma humbertiana	India, Andhra Pradesh	JQ907155	JQ906985	JQ907048	JQ907102
	Oligotoma nigra	USA, Arizona	JQ907138	AY125274	N/A	AY125221
	Oligotoma saundersii	Trinidad and Tobago	EU157039	EU157051	EU157062	EU157031
Teratembiidae	*Teratembia geniculata*	Argentina, Chaco	JQ907150	JQ906977	JQ907040	JQ907099
	Teratembia sp. KBMEB7	Brazil, Gurinhem	JQ907141	JQ906968	JQ907031	JQ907094

Supplementary Table S2. Primers used in this study.

Primer name	Primer sequence
CZTF-ND5-10F	AAATGGCAACAAATCCCGAAAC
CZTF-ND5-10R	TTTGTAGATGGTTGCCGATTGC
CZTF-COB-10F	CGATTCTTCTCATTCCATTTTATTTT
CZTF-COB-10R	TAATTATGGGGTTAGCGATGGAAA
CZTF-CO1-10F	GACACCCGAGCATATTTTACATCA
CZTF-CO1-11R	AGGAAGTCGTTCAAGTGTTATGTTTAT
CZTF-16S-10F	TATCTATCACCCCAATAAAATCCAAT
CZTF-16S-3R	ATGGCTGCGGTAATTTGACTGTGT
CZTF-ND2-T1F	ATCAACACCATATCTTTCATCCCTT
CZTF-ND2-10R	AGTCCTTGTATTACGTTTGTAAATCAA
CZTF046-1F	TGGTTTATCTTTGTTGGTTATTATAG
CZTF046-2R	ATTCAATCTCACTATTACAACACCA
CZTF048-1F	TTGATTTCTTCTGTTGTTTTAACTT
CZTF048-2F	GGGATGAGTGGTTTCCTTGA
CZTF048-3R	TTCTAAACAACCTATTACAACATTTG
CZTF056-1R	TTTACCCTAAACCCATAAATAAATT

CZTF088-1F	AATTTCAACTTTGAAGGTTAATAGTT
CZTF104-1F	TTAACATTTCACTCCCAAGAA
CZTF104-2F	TGGCACATATTTAACCCCTAA
CZTF104-3R	ATTTGGATTAGGAGTTATTGG
